# Cerebral microbleeds: overview and implications in cognitive impairment

**DOI:** 10.1186/alzrt263

**Published:** 2014-06-11

**Authors:** Sergi Martinez-Ramirez, Steven M Greenberg, Anand Viswanathan

**Affiliations:** 1 Philip J. Kistler Stroke Research Center, Massachusetts General Hospital, 175 Cambridge Street Suite 300, Boston, MA 02114, USA

## Abstract

Cerebral microbleeds (MBs) are small chronic brain hemorrhages which are likely caused by structural abnormalities of the small vessels of the brain. Owing to the paramagnetic properties of blood degradation products, MBs can be detected *in vivo* by using specific magnetic resonance imaging (MRI) sequences. Over the last decades, the implementation of these MRI sequences in both epidemiological and clinical studies has revealed MBs as a common finding in many different populations, including healthy individuals. Also, the topographic distribution of these MBs has been shown to be potentially associated with specific underlying vasculopathies. However, the clinical and prognostic significance of these small hemorrhages is still a matter of debate as well as a focus of extensive research. In this article, we aim to review the current knowledge on the pathophysiology and clinical implications of MBs, with special emphasis on the links between lobar MBs, cerebral amyloid angiopathy, and Alzheimer’s disease.

## Introduction

Cerebral microbleeds (MBs) are small chronic brain hemorrhages, likely caused by structural abnormalities of the small vessels. The paramagnetic properties of blood degradation products make possible the visualization of MBs *in vivo*, using specific magnetic resonance imaging sequences. Extensive research has demonstrated the value of MBs as markers of small-vessel disease. Indeed, specific topographic patterns of MBs are thought to be representative of particular underlying vasculopathies, mainly cerebral amyloid angiopathy and hypertensive vasculopathy. As such, MBs are regularly identified in individuals from stroke and memory clinics, where they might have implications in therapeutic management. Interestingly, MBs are also a common finding in other populations, even in healthy elderly individuals. The clinical and prognostic significance of MBs in all these settings remains poorly understood. In this review, we aim to summarize the current knowledge on the pathophysiology and clinical implications of MBs, with special emphasis on the links between lobar MBs, cerebral amyloid angiopathy and Alzheimer’s disease.

## Review

### Description and epidemiology

**Figure 1:**
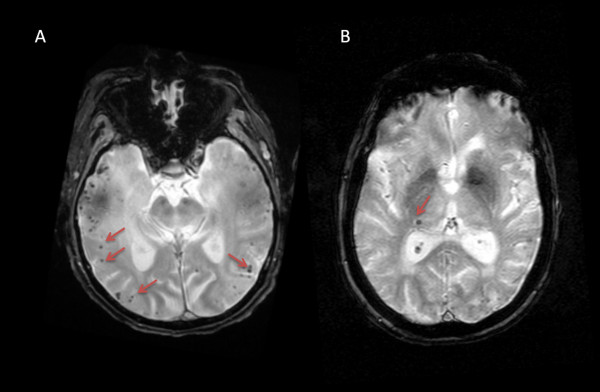
**Cerebral microbleeds as seen on magnetic resonance imaging gradient-recalled echo imaging (arrows). (A)** Multiple lobar microbleeds distributed across the temporal lobes. **(B)** Isolated deep microbleed in the lateral aspect of the right thalamus.

From a pathological point of view, MBs are tiny deposits of blood degradation products (mainly hemosiderin) contained within macrophages and in close spatial relationship with structurally abnormal vessels. Hemosiderin is a strong paramagnetic material, which allows its detection when a magnetic field is applied [1]. This phenomenon, called susceptibility effect, is the basis of T2*-gradient recalled echo (GRE) imaging, which led to the definition of the current concept of radiological MBs [2] (Figure 1). Over time, further sequences have been developed, including three-dimensional T2*-GRE [3] and the most sensitive one to date - susceptibility-weighted imaging (SWI) [4]. Furthermore, the upgrade of several MRI parameters, such as the magnetic field, has also contributed to a more sensitive detection of MBs [5,6]. For instance, 7-Tesla MRI detects twice as many MBs in comparison to conventional 1.5-Tesla MRI [7]. The downsides of these technical improvements are the increase in the ‘blooming effect’ (larger visual appearance of MBs on MRI than the actual size of the hemosiderin deposit) [8] and the frequency of MB mimics, which raises concerns about potential ‘overdetection’ of MBs and a limited clinical significance (especially if supporting pathological data are not available). Also, the variation of parameters causes difficulties for a unified definition of MBs. Still, consensus guidelines on MB detection and interpretation have been published [9]. Based on these guidelines, MBs can be described as small areas of signal void with associated ‘blooming’, excluding non-hemorrhagic causes of signal void. Concerning MB size, a study on hemorrhage volumes in patients with cerebral amyloid angiopathy (CAA) found a bimodal distribution, instead of a continuum, with a large gap between the two peaks representing MBs and macrobleeds. This argued against setting a strict limit for the maximum diameter of MBs; however, the study reported a value of 5.7 mm as the best cutoff to distinguish between the two types of hemorrhages [10].

MBs were first reported in association with intracerebral hemorrhage (ICH) [11]. After this initial study, reports of MBs in ICH and other populations have dramatically increased. As the frequency of MBs varies enormously depending on the MRI study characteristics and the selection of the study subjects, the reported prevalence in different clinical conditions has considerably wide ranges: 47% to 80% in ICH [12,13], 18% to 71% [12,14] in ischemic stroke, or 17% to 46% in cognitive decline/dementia [15]. However, even given the lowest estimates, it appears that MBs are a common phenomenon across different patient populations. On the other hand, several population-based studies have also reported on MB prevalence in healthy older individuals, which can be as high as 23.5% [16]. This observation raises questions about the pathological significance of MBs and the importance of MB detection in asymptomatic individuals.

### Pathological significance

Neuroimaging studies have consistently reported associations between MB, vascular risk factors (age and hypertension) and previously well-established markers of small-vessel disease (SVD), such as lacunar infarcts and white matter hyperintensities (WMHs) [17]. Also, a high frequency of MBs in severe vascular conditions like ischemic and hemorrhagic stroke has been noticed [17]. Taken together, these observations strongly support MBs as an additional marker of SVD.

A few small histopathological studies have provided insight into the vascular anomalies associated with MBs [8,18-21]. In these studies, two main forms of vasculopathies have been associated with MBs in the aging brain: CAA and hypertensive vasculopathy (HV). CAA is caused by the accumulation of β-amyloid on the vessel walls of cortical and leptomeningeal arteries. HV, a consequence of long-standing hypertension over the microvasculature of the brain, is pathologically defined by the presence of lipofibrohyalinosis, which affects mostly the deep penetrating arterioles. As both entities are associated with age, they may coexist in a single individual, with variable degrees of severity [8].

Because of the differential topographic preference of CAA and HV, MBs associated with these two entities could be expected to follow similar distributions: strictly lobar (cortical-subcortical regions of brain lobes and cerebellum) in CAA; strictly deep (deep white matter, basal ganglia, thalamus, brainstem, cerebellum) in HV; and mixed (lobar and deep regions) when an individual has coexisting CAA and HV. However, there is no definitive evidence supporting a high diagnostic value of these MB patterns for CAA/HV. First, the aforementioned histopathological studies consisted of small series providing very limited observations, especially regarding lobar MBs and CAA. Second, direct extrapolations from the Boston Criteria for the diagnosis of CAA-related hemorrhage [22] (Table 1) seem inadequate, as they have been validated only in subjects with lobar ICH. At present, it is indirect evidence from population-based studies that mostly supports the associations between lobar/deep MBs and CAA/HV. The Rotterdam Scan Study [16] reported that healthy older individuals with strictly lobar MBs have an exceedingly high frequency of the apolipoprotein E-ϵ4 (APOE-ϵ4) allele (compared with patients with MBs not strictly confined to lobar regions), which is in agreement with increased APOE-ϵ4 frequencies seen in patients with ‘probable CAA’. In contrast, strictly deep MBs were associated with vascular risk factors, lacunar infarcts, and WMH, but not with the APOE-ϵ4 allele. Associations of mixed MBs resembled the profile of strictly deep MBs. In a subsequent study based on the same population, lobar MBs were seen to occur significantly more often in the temporal lobe [23], one of the regions severely affected by CAA. There still exists another line of investigation providing support to the link between lobar MB and CAA, and it consists of the study of CAA patients with both MRI and Pittsburgh compound B (PiB)-positron emission tomography (PET) imaging. With this combined approach, a close spatial relationship between MBs and vascular amyloid load was found in a cross-sectional study [24]. Further supporting this observation, PiB retention was shown to rapidly decrease with increasing distance from the MB site [24]. In a later study on a smaller cohort with longitudinal data, the investigators concluded that high-load amyloid areas are a preferential site for development of incidental lobar hemorrhages [25]. Neuroimaging-pathological correlation studies are needed to confirm these associations.

**Table 1:** **Boston Criteria for diagnosis of cerebral amyloid angiopathy-related hemorrhage **[22]

1. Definite CAA	Full post-mortem examination demonstrating:
	• Lobar, cortical, or corticosubcortical hemorrhage
	• Severe CAA with vasculopathy^a^
	• Absence of other diagnostic lesion
2. Probable CAA with supporting pathology	Clinical data and pathologic tissue (evacuated hematoma or cortical biopsy) demonstrating:
	• Lobar, cortical, or corticosubcortical hemorrhage
	• Some degree of CAA in specimen
	• Absence of other diagnostic lesion
3. Probable CAA	Clinical data and magnetic resonance imaging (MRI) or computed tomography (CT) demonstrating:
	• Multiple hemorrhages restricted to lobar, cortical, or corticosubcortical regions (cerebellar hemorrhage allowed)
	• Age >55 years
	• Absence of other cause of hemorrhage^b^
4. Possible CAA	Clinical data and MRI or CT demonstrating:
	• Single lobar, cortical, or corticosubcortical hemorrhage
	• Age >55 years
	• Absence of other cause of hemorrhage^b^

Criteria were established by the Boston Cerebral Amyloid Angiopathy Group: Steven M Greenberg, Daniel S Kanter, Carlos S Kase and Michael S Pessin. ^a^As defined in [26]. ^b^Other causes of intracerebral hemorrhage were excessive warfarin (international normalized ratio (INR).3.0); antecedent head trauma or ischemic stroke; central nervous system tumor, vascular malformation, or vasculitis; and blood dyscrasia or coagulopathy. (INR.3.0 or other non-specific laboratory abnormalities are permitted for diagnosis of possible cerebral amyloid angiopathy).

### Clinical implications

Direct pathological observations have demonstrated the existence of tissue damage surrounding MBs [7,15-17]. On a less direct level, diffusion tensor imaging studies have shown an independent association between the presence of MBs and a higher degree of microstructural injury of the brain [27,28]. These phenomena provide a scientific basis to support direct clinical effects of MBs, beyond their associations with particular vasculopathies.

Although the underlying mechanism is still a matter of debate, several clinical reports suggest that MBs might cause acute transient focal neurological episodes (TFNEs) [29,30]. Clinically, these episodes may resemble transient ischemic attack (TIA) or seizures, depending on the negative or positive character of the symptoms. Pathogenesis might involve direct damage to cells/tracts, but electrical disturbances associated with the leakage of blood components may have a more significant role. In fact, experimental studies have shown that MBs may transiently affect the function of the nearby cells because of an inhibition of stimulus-evoked calcium responses [31]. Recent studies are pointing more toward superficial cortical siderosis, instead of MBs, as the main CAA feature associated with TFNE. Regardless of the exact type of lesion involved, the investigation for evidence of chronic hemorrhages in TIA seems crucial, as the simple initiation of anti-thrombotic therapy could have undesirable effects in cases with TFNE.

The cautious approach to anti-thrombotic therapy in patients with these symptomatic episodes can be extended to all patients exhibiting MBs. From a pathophysiological standpoint, MBs appear to be the expression of a hemorrhage-prone state of the brain, which might carry a greater risk of ICH. In the literature, the risk/benefit ratio of anti-thrombotic drugs in individuals with MBs is controversial, and no formal contraindications in this respect exist. Still, some data support the presence of MBs as an independent risk factor for warfarin-related ICH [32]. Even anti-platelet agents, traditionally safer than anti-coagulants, have been associated with an increased risk of ICH, especially in subjects with a high number of MBs [33,34]. Given these observations, it seems reasonable to individualize decisions on anti-thrombotic therapy in patients with MBs.

A few longitudinal studies have investigated the progression of MBs over time, revealing that MBs at baseline are a risk factor for the development of new MBs [35-37]. According to a follow-up report from the Rotterdam Scan Study, incident lobar and deep MBs have different risk factors [35], similar to what had been observed with baseline MBs [16]. The importance of cumulative MB burden is double: first, it may produce further widespread damage over brain structures; and, second, it highlights the progression of the underlying SVD. These two factors may explain the impact of baseline identification of MBs on future neurological events and mortality. In a study of individuals with lobar ICH, a higher number of lobar hemorrhages at baseline (including MBs) predicted an increased risk of not only lobar ICH recurrence but also cognitive decline, functional dependence, or death in those individuals not dependent or demented by the time of admission [38]. Mortality was also strongly predicted by MBs (especially when multiple) in another study following patients in a large memory clinic cohort [39]. When specific causes of death according to MB distribution were investigated in a population-based cohort of older people at high risk of cardiovascular disease, deep MBs were associated with cardiovascular mortality, whereas lobar MBs were associated with stroke-related mortality [40]. These findings fit well with the notion of lobar and deep MBs associated with HV and CAA, respectively. Since HV is secondary to a systemic process (hypertension), high cardiovascular mortality is expected in the context; however, CAA is a primary brain vasculopathy, with no extracerebral manifestations.

Apart from ICH, the other main neurological outcomes that have been associated with MBs are gait disturbances [41,42] and cognitive impairment [43]. Cognitive impairment (and dementia) represents an increasing source of severe long-term disability and will be the focus of the review in the next sections.

### Microbleed and cognitive impairment

One of the initial studies assessing the cognitive impact of MBs compared the performance on multiple cognitive domains between patients with and without MBs from a neurovascular clinic [44]. The two subgroups were matched for age, gender, intelligence quotient, extent of WMH, and type and location of ischemic stroke. Individuals with MBs had a much higher prevalence of executive dysfunction than those without MBs (60% versus 30%, *P* = 0.03). In logistic regression analyses, the presence of MBs was the only independent predictor of executive dysfunction. Interestingly, in individuals with executive dysfunction, MBs were predominantly located in the frontal lobes and basal ganglia, areas classically considered the neuroanatomical substrate for executive function. These results suggested that (a) MBs may actually have a negative effect on cognition, independently of other concurrent vascular lesions, and (b) there seems to be an anatomical correlation between the distribution of MBs and the cognitive domains affected, suggesting a direct damage of MBs over the tissue as the pathogenic mechanism. Later studies have confirmed and expanded these findings, using different study populations with different MB patterns. Seo and colleagues [45] investigated the independent effect of MBs in multiple domains in a cohort of individuals with diagnosed subcortical vascular dementia. MBs were predictive not only of executive dysfunction but also of memory, language, and visuospatial impairment. MBs were distributed mostly in the cortical areas, predominantly in the fronto-temporal lobes, and this might suggest a high prevalence of CAA in this cohort. Again, this predominant MB location matched well with the impaired cognitive areas. In the context of Alzheimer’s disease (AD), several studies have also explored the relationship between MBs and cognition. We discuss this complex interplay between lobar MBs, AD, and CAA in detail below.

Both the Age, Gene/Environment Susceptibility (AGES)-Reykjavik study and the Rotterdam Scan Study have reported on MBs and cognitive performance in their respective population-based cohorts. The AGES study (n = 3,906) [46] showed that the presence of MB, especially multiple MBs, is associated with worse processing speed and executive function. These results were stronger in subjects with strictly deep MBs. It was also seen that the combination of multiple MBs and retinopathy increased the odds ratio of vascular dementia: 3.10; 95% confidence interval (CI) 1.11 to 8.62 [46]. Taken together, these data firmly provide support that microvascular damage plays a key role in cognitive impairment in older individuals living in the community. Whereas the AGES study confirmed in community-dwelling individuals the suggested link between deep MBs and subcortical cognitive deficits, the Rotterdam Scan Study emphasized the negative effects of lobar MBs on a wider spectrum of cognitive domains [43]. In this study of 3,979 participants, multiple MBs (at least five) were associated with worse cognitive performances in all domains but memory. However, these associations were more robust in individuals with strictly lobar MBs (all analyses adjusted for age, sex, education, vascular risk factors, other SVD markers, and brain atrophy). Differences in baseline characteristics between these two population-based cohorts may explain why deep or lobar location of MBs appears to be more prominent.

In general, the available literature provides support that MBs are independent contributors to cognitive impairment and that their topographic distribution may have specific associations with certain cognitive domains. As stated, direct tissue damage or underlying SVD (or both) may account for these detrimental effects.

#### Lobar microbleed, cerebral amyloid angiopathy, and Alzheimer’s disease

MBs have extraordinary importance in the context of AD. Apart from offering hints on AD pathophysiology, their presence may modify the course of the disease and even the response to new immunotherapeutic agents.

The frequency of MBs in subjects with AD varies significantly across studies (16% to 32%) [15,47-50], with a pooled proportion of 23% (95% CI 17% to 31%) [51]. Despite this high variability, MB overall prevalence is consistently higher in subjects with AD than in non-demented, older individuals [15,50]. Indeed, a recent study using high-field MRI found an MB prevalence as high as 78% in patients with early AD [7]. Although deep MBs may be identified in some AD cases, the vast majority of them (92%) show a lobar predominance. As pointed out in population-based studies, lobar MBs are not associated with classic vascular risk factors and show weak associations with other classic SVD markers. Because CAA is present in up to 90% of AD cases [52], it may be conceptually feasible to state that lobar MBs are reliable markers of CAA in patients with AD. The ‘amyloid cascade’ hypothesis [53], in combination with further theories on amyloid clearance through perivascular spaces [54], supports this notion. However, it is important to note that only a small proportion of AD cases (23%) actually exhibit lobar MBs [51]. There are several ways to explain this dissociation between the post-mortem pathological findings of CAA and MB detection during life. First, lobar MBs may appear only in cases with advanced CAA, and advanced CAA accounts for only around 25% of individuals with dementia [55]. Also, CAA is often reported in autopsies, which by definition reflect end-stage disease, whereas MB imaging is performed mostly in earlier stages of the disease. Third, the implementation of more sensitive MRI sequences for MB detection will probably increase the proportion of AD patients with lobar MBs.

Despite this, there is a possibility that AD patients with lobar MBs represent a subgroup with distinct characteristics. This concept has been studied by comparing the cognitive profile, the rate of cognitive decline over time, and the mortality rates between MB and non-MB subjects with AD. Two early studies failed to demonstrate any influence of MBs on cognitive performance in AD cohorts [47,49]. The main limitation of these studies was the use of the Mini-Mental State Examination (MMSE) as the main cognitive outcome measure. Indeed, global cognitive tests (like MMSE) may not capture impairment in certain domains such as executive function. However, a larger study using specific neuropsychological assessments did not find any relationship between MBs and worse cognitive performance [50]. In this case, low MB counts may have prevented this study from identifying associations. More recently, another study overcame this issue by comparing multiple MB cases with non-MB cases within an AD cohort. This study showed that AD subjects with multiple MBs had a more severe cognitive impairment (independently of disease duration) and degree of atrophy and WMH [56]. Two studies investigated the value of MBs in predicting progression from mild cognitive impairment to dementia. One of these studies found that the presence of at least one MB yielded a more than twofold increase, but not a significant risk of non-AD dementia [57]. In the other study, MBs detected on SWI sequences were found to predict cognitive decline in patients followed up to 5 years [58]. Although data are very limited, it is conceivable that lobar MBs could predict progression to AD-type dementia but that deep MBs could anticipate the future development of vascular dementia. In terms of mortality, a study showed that the presence of MBs at baseline in patients from a memory clinic was associated with an increased risk of death, in a dose-dependent fashion and independently of other SVD markers and vascular comorbidity [39]. A later study on the same cohort reported that MBs were not associated with a faster rate of cognitive decline, suggesting that the increase in mortality may be related to other clinical events, like ICH [59].

Finally, MBs may have some impact on current immunotherapies for AD. An early trial of active immunization reported some cases of severe meningoencephalitis, which prompted its termination [60]. The pathologic study of one of these cases [61] suggested that an inflammatory reaction had been triggered by the immunization agent and targeted β-amyloid, both in tissue plaques and vessels [62]. Consequently, the presence of advanced CAA has been established as a potential risk factor for developing undesirable brain inflammation in AD immunotherapy. Since lobar MBs in the context of AD are interpreted as markers of advanced CAA, lobar MB carriers (especially those with multiple MBs) are currently excluded from immunization trials as a safety measure [63]. Although this seems to be a reasonable approach, the precise correlation between MB burden and CAA presence (and severity) is still unknown.

## Conclusions

MBs are SVD markers that carry diagnostic and prognostic information for individuals in various clinical settings. Although our knowledge on MB pathophysiology and clinical implications has increased substantially in the last decades, important questions remain unanswered. The implementation of more sensitive MRI techniques for the detection of MBs, and their systematic assessment along with other imaging markers (including PET-based amyloid imaging [24]) and blood biomarkers, may provide a useful tool in the future to guide therapeutic decisions and better define subjects in a research context.

## Abbreviations

AD: Alzheimer’s disease; AGES: Age, Gene/Environment Susceptibility; APOE-ϵ4: apolipoprotein E-ϵ4; CAA: cerebral amyloid angiopathy; CI: confidence interval; GRE: gradient recalled echo; HV: hypertensive vasculopathy; ICH: intracerebral hemorrhage; MB: microbleed; MMSE: Mini-Mental State Examination; MRI: magnetic resonance imaging; PET: positron emission tomography; PiB: Pittsburgh compound B; SVD: small vessel disease; SWI: susceptibility-weighted imaging; TFNE: transient focal neurological episode; TIA: transient ischemic attack; WMH: white matter hyperintensity.

## Competing interests

SMG is the principal investigator in the following grants related to CAA: title: Amyloid Angiopathy, sponsor: National Institutes of Health-National Institute on Aging (NIH-NIA), sponsor number: 5R01AG026484; title: Early Detection of CAA, sponsor: NIH-NINDS, sponsor number: 5R01NS070834. AV is the principal investigator in the following grants related to CAA: title: Project II, sponsor: NIH-NIA, sponsor number: 5P50AG005134; title: Effect of WMD on Gait and Balance in CAA, sponsor: NIH-NIA, sponsor number: 5K23AG028726. SM-R declares that he has no competing interests.
